# Anti-Müllerian Hormone: A Predictor of Successful Intrauterine Insemination

**DOI:** 10.7759/cureus.47200

**Published:** 2023-10-17

**Authors:** Allison Stalzer, Dara Seybold, Pickens Gantt, Mike Broce, Ashley Cronkright

**Affiliations:** 1 Obstetrics and Gynecology, Charleston Area Medical Center/West Virginia University, Charleston, USA; 2 Institute for Academic Medicine, Charleston Area Medical Center, Charleston, USA; 3 Internal Medicine, Charleston Area Medical Center/West Virginia University, Charleston, USA

**Keywords:** pregnancy, ovarian stimulation, intrauterine insemination, female infertility, anti-mullerian hormone

## Abstract

Introduction: The anti-Müllerian hormone (AMH) produced by the granulosa cells of ovarian follicles has been shown to correlate with ovarian reserve and is often measured for fertility therapies. In this study, we evaluated the relationship between serum AMH values and the clinical pregnancy (CP) rates of female partners with unexplained infertility undergoing intrauterine insemination utilizing varying ovarian simulation protocols.

Methods: This is a retrospective cohort study conducted among couples who underwent intrauterine insemination therapy over a period of four years at Charleston Area Medical Center, a tertiary care medical center in West Virginia, USA. Logistic regression was used to determine the best predictor of CP.

Results: A total of 509 intrauterine inseminations resulting in 81 (15.9%) Cps were analyzed. The cycles with a CP had higher mean AMH values (3.7+3.5 vs. 2.2+2.1; p<0.001). The majority of patients were nulliparous (77.0%) with a mean age of 33.6+5.0 years. After including only patients with unexplained infertility (the predominate infertility diagnosis; n=255 (50.1% of the cycles)) and stimulation cycles >10, the final sample size for the analysis was 245/509=48.1%. Following a receiver operating characteristic (ROC) curve analysis, the optimal AMH cut-off point was 2.1 ng/mL with an area under the curve (AUC) equal to 0.61 and 95% confidence intervals (CIs) of 0.55- 0.67 (p=0.002). The CP rate was significantly higher with the AMH >2.1 ng/mL (20.0%) compared to <2.1 ng/mL (10.0%; p=0.041). With Clomid/human gonadotropins/human chorionic gonadotropin (hCG) trigger treatment, the CP rate quadrupled (odds ratio (OR): 4.6; 95% CI: 2.1-9.7; p<0.001).

Conclusion: This study indicates that higher AMH levels and a more aggressive ovarian stimulation protocol for intrauterine insemination therapy (IUI) have a better probability of resulting in CP.

## Introduction

Testing ovarian reserve is a part of evaluating a woman for fertility treatment. Testing methods primarily include cycle day three follicle-stimulating hormone (FSH) or estradiol (E2), clomiphene citrate challenge test, and anti-Müllerian hormone (AMH) or antral follicle count (AFC). While none of these tests or combinations have proved to be superior, AMH is considered more convenient and reliable than FSH and E2 because AMH values do not vary during the menstrual cycle; therefore, it can be obtained on any cycle day [1]. In addition, AMH does not have the confounding issue of operator discretion that is characteristic of basal transvaginal AFC [2].

AMH is produced by granulosa cells of the female ovarian follicles, and levels decline with maternal age [3]. A diminished ovarian reserve correlates with a significantly lower probability of achieving a clinical pregnancy (CP). While AMH has been shown to correlate with ovarian reserve in candidates for fertility treatment, these values can provide an indicator of expectations and management of treatment [4-6]. In support of research indicating that AMH is more specific than FSH, a study reported that AMH <1.05 ng/mL does not define diminished ovarian reserve but rather defines a significantly decreased chance of live birth [7]. In another study on the association of AMH and oocytes retrieved during assisted reproductive technology (ART), higher day three serum AMH concentrations were associated with a greater number of oocytes retrieved [8]. Several studies have been published on AMH and in vitro fertilization (IVF), showing a correlation between AMH levels and ovarian response to stimulations [9-13]. However, the utility of AMH in the setting of intrauterine insemination therapy (IUI) has not been adequately studied. One study retrospectively analyzed 811 IUI cycles with clomiphene citrate and HMG and had an overall CP rate of 12.6% per cycle [14]. The number of follicles proved to be an important predictor of IUI outcome with the highest CP rate seen in the presence of three follicles [14]. In another study of 243 individuals undergoing IUI with 28 (13.3%) achieving a live birth after one cycle of IUI, AMH better predicted outcome than FSH or AFC [15]. However, some studies of patients undergoing IUI did not find AMH as a predictor of CP or live birth [2,16-20]. In one study that examined infertility in women <38 years, researchers determined that young women who had accelerated follicular depletion, as evidenced by either lower AMH levels or post-cycle low oocyte yield, had similar rates of blastulation and live birth when compared to matched controls with normal AMH levels and post-cycle parameters [21]. In addition, other factors affecting fecundity have been researched, including one study comparing double IUI, performed 18 and 36 hours following human chorionic gonadotropin (hCG) or the day of and day following LH surge versus single IUI, performed at 36 hours following hCG or the day following LH surge, with greater success rates seen in the double IUI group [22].

More recently, however, studies do continue to show a positive correlation between AMH levels and oocyte quality. This correlation remained clinically significant even in patients with infertility issues, such as in polycystic ovary syndrome (PCOS). Moreover, when age-stratified AMH was used in the model analysis, combined alongside beta endorphin (β-EP) levels, there was a significant positive correlation with CP rates and live births after IVF [23]. Another study attempted to evaluate whether AMH correlates with oocyte quality in women with advanced age. Women over the age of 37 were divided into AMH high- and low-level groups, with a cutoff of 1.1 ng/ml. The study found that, compared with women in the high-AMH group, women in the low-AMH group had similar rates of fertilization and blastocyst formation. While the rates of implantation and live births were similar between the groups, the pregnancy rate was reduced in the low AMH group, most likely due to the number of high-quality embryos that were transferred in each cycle [24]. With the aim of adding to the literature, the purpose of the study was to further evaluate the relationship between serum AMH values and CP rates of female partners with a diagnosis of unexplained infertility utilizing varying ovarian stimulation protocols.

## Materials and methods

Study design

This original research was reviewed and approved by the local institutional review board of Charleston Area Medical Center/West Virginia University - Charleston Division (approval no. 1997132) with informed consent waived. This study is a retrospective cohort study. The study population is composed of women who have been receiving IUI therapy over a period of four years at Charleston Area Medical Center, a tertiary care medical center in West Virginia, USA, and AMH levels were analyzed.

Selection criteria

Data from IUIs were retrieved and entered into the study proforma from the clinical records of clients that had treatments for infertility. This included natural (unstimulated) cycles and ovarian stimulation protocols utilizing clomiphene citrate and/or gonadotropins. Patients’ menstrual cycles were monitored using basal body temperature charts and hCG trigger was planned 36 hours prior to the IUI procedure. Transvaginal ultrasounds were used for follicle monitoring during the stimulation protocol. Clinical and laboratory parameters associated with CP rates were recorded, including AFC. The patients were categorized into two groups: diagnosed infertility and unexplained infertility. IUI and patient characteristic were compared between the two groups. For the second part of the analysis, we excluded patients with defined infertility (including PCOS, which is also associated with elevated levels of AMH). Only IUI data from patients with unexplained infertility and stimulation protocols with 10 or more cycles were analyzed, comparing AMH and AFC values for pregnancy outcome (CP versus no CP). A cut-off point for AMH to predict CP was calculated, and then the best predictor of CP was determined by including all statistically significant variables at the univariate analysis.

Statistical analysis

Data were analyzed using IBM SPSS Statistics for Windows, version 19 (released 2010; IBM Corp., Armonk, New York, United States). Continuous variables were presented as means and standard deviations and were compared using Student's independent samples t-tests, while categorical variables were reported as percentages and compared using a chi-squared or Fisher’s exact test to determine if there was an association between AMH levels, AFC >16, and CP rates. An alpha of 0.05 was set in the determination of statistical significance.

Receiver operating characteristic (ROC) curves were used to determine the cut-off point in AMH levels and AFC to predict CP. To identify the cut-off value, we calculated the Youden index (*J*) using the formula (*J* = maximum sensitivity + specificity -1), which is defined as the maximum vertical distance between the ROC curve and the diagonal line or the point on the curve farthest from chance [25]. Backward stepwise logistic regression was used to determine the significant predictors of CP and included the following potential variables: maternal age, AMH, AFC, and stimulation protocol. All potential variables were entered into the full model, and then the least and non-significant variables were removed from each step until only the significant predictors remained in the final reduced model. A logistic regression model was used to determine the OR and CI for the significant predictor variables.

## Results

A total of 509 IUIs with 81 (15.9%) CPs from 254 female partners whose serum AMH was assesed was identified for potential inclusion in this study. Of the 509 total IUIs, cycles resulting in CPs had higher mean AMH values (3.7+3.5 vs. 2.2+2.1; p<0.001). The majority were nulliparous (77.0%) with a mean age of 33.6+5.0 years. Unexplained infertility was the predominant diagnosis for 50.1% of the patient cycles (n=255), followed by PCOS constituting 20.6% (n=105) and azoospermia (9.0%; n=46). The mean AMH value was 3.0+3.0 ng/mL, and the mean AFC was 3.27 +1.9. Table 1 presents in detail the patient characteristics.

**Table 1:** Intrauterine insemination characteristics IUI: intrauterine insemination; AMH: anti-Müllerian hormone; PCOS: polycystic ovarian syndrome

	Total IUI cycles(n=509)	Patients with both unexplained infertility and IUI cycles >10 (n=245)	Diagnosed infertility or IUI cycles <10 (n=264)	p value
Maternal age (years)	33.6 ± 5.0	33.9 ± 5.2	33.4 ± 4.7	0.241
Number of follicles	3.27 ± 1.9	3.3 ± 1.8	3.2 ± 2.0	0.363
AMH value (ng/mL)	3.0 ± 3.0	2.2 ± 2.1	3.7 ± 3.5	<0.001
Parity				0.096
Nulliparous	392 (77.0%)	182 (74.3%)	210 (79.5%)	
Multiparous	117 (23.0%)	63 (25.7%)	54 (20.5%)	
Hypertension	38 (7.5%)	10 (4.1%)	28 (10.6%)	0.006
Thyroid condition	35 (6.9%)	21 (8.6%)	14 (5.3%)	0.163
Diabetes	10 (2.0%)	4 (1.6%)	6 (2.3%)	0.753

**Figure 1:**
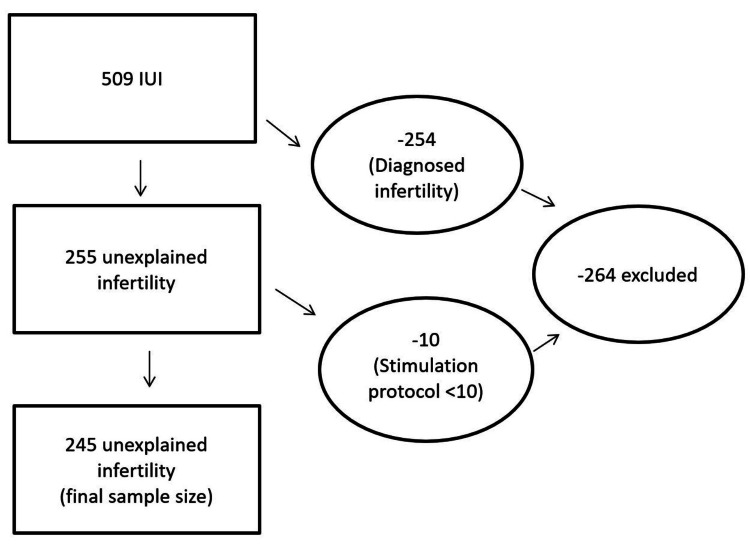
A flow diagram of the intrauterine insemination cycles evaluated

There were only two stimulation protocols with 10 or more cycles: (1) Clomid with an hCG trigger injection (ChCG) and (2) Clomid, human gonadotropins, and an hCG trigger injection (ChmghCG). Thus, the final sample size for the analysis was 245/509=48.1% of the IUIs as these were female partners with unexplained infertility with 10 or more cycles for the ovulation stimulation protocol. Figure 1 provides more details of how the included IUI cyles were determined.

**Figure 2:**
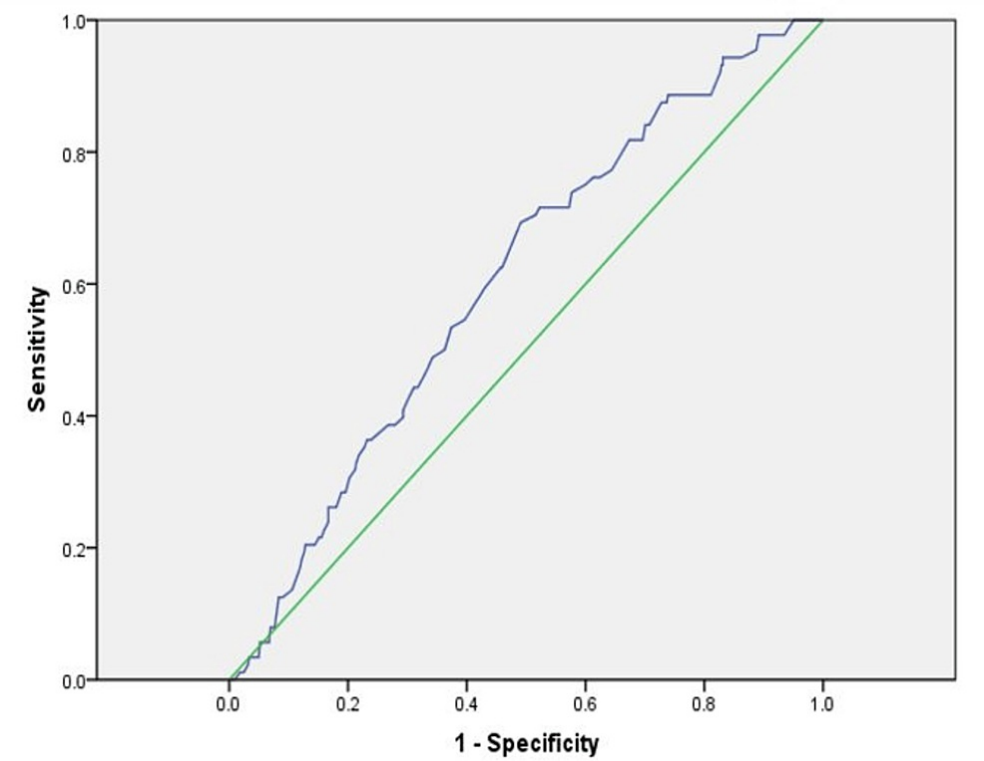
Receiver operating characteristic curve cut-off to predict clinical pregnancy

In the final sample size of 245 IUIs, the AMH values were significantly higher, i.e., 2.9+2.4 ng/mL versus 2.1+2.1 ng/mL in the CP versus the no-CP group (p=0.044) for patients with unexplained infertility. Likewise, we found a significantly higher AFC, i.e., 4.0+2.0 versus 3.2+1.7 in the CP versus no-CP group (p=0.018). Following the ROC analysis, the optimal AMH cut-off point was 2.1 ng/mL with an area under the curve (AUC) equal to 0.61 and 95% CI of 0.55-0.67 (p=0.002), while the cut-off value for AFC was 3.5 (AUC: 0.61; p<0.002; Figure 2), indicating some concordance for both.

The CP rate was significantly higher for IUI cycles where female partners had an AMH value >2.1 ng/mL (21/105; 20.0%) compared to <2.1 ng/mL (14/140; 10.0%; p=0.041). There was a higher CP rate for ChmghCG ovarian stimulation (25/140; 17.9%) as opposed to ChCG (10/105; 9.5%; p=0.068) although not statistically significant. However, when the stimulation protocol was cross-tabbed with the AMH cut-off, cycles with ChmghCG and AMH >2.1 ng/mL had significantly more success with CP, i.e., 17 (32.1%) compared to <2.1 ng/mL 8 (9.2%; p=0.001), as expressed in Table 2.

**Table 2:** Clinical pregnancy in relation to the AMH level and stimulation protocol in IUI cycles in patients with both unexplained infertility and IUI cycles >10 (n=245) ChCG: Clomid + hCG trigger; ChmghCG:Clomid + human gonadotropins + hCG trigger; AMH: anti-Müllerian hormone; IUI: intrauterine insemination

Stimulation protocol	Clinical pregnancy	AMH <2.1 ng/mL	AMH >2.1 ng/mL	p value
ChCG	No	47 (88.7%)	48 (92.3%)	0.741
	Yes	6 (11.3%)	4 (7.7%)	
ChmghCG	No	79 (90.8%)	36 (67.9%)	0.001
	Yes	8 (9.2%)	17 (32.1%)	

After the multivariate analysis, an association was found between an AMH>2.1 ng/mL and ChmghCG treatment. IUI cycles where female partners with an AMH value >2.1 ng/mL who received a ChmghCG treatment were over four times more likely to have a CP (OR: 4.6; 95% CI: 2.1-9.7; p<0.001).

## Discussion

Evaluating ovarian reserve by examining AMH values before fertility treatment allows for an alteration of the treatment method should a patient have an inadequate ovarian reserve. We found that AMH levels >2.1 ng/mL demonstrated an increased ovarian reserve with a higher CP rate of 20.0%. With the results of our study of 245 IUI cycles, we noted that an AMH level of >2.1 ng/mL had a significantly higher rate of success with IUI with a stimulation protocol rate of 32.1% with the ChmghCG protocol compared to 9.2% with AMH <2.1 ng/mL with the same stimulation protocol (p=0.001). 

While a cut-off value for increased ovarian reserve has not yet been determined in the literature, our study suggests that a higher AMH may indicate a better probability of a live birth. Our results are very similar to the results of a meta-analysis conducted with 19 studies (n=4324) on AMH as a predictor of implantation and/or CP in women undergoing assisted reproductive technology [26]. In this meta-analysis, the OR was 2.10 (95% Cl 1.82-2.41) with an AUC of 0.634 (95% CI0.618-0.650) [26]. Another similar study had an AUC of 0.53 at the first attempt with a positive correlation between AMH levels and CP [27]. A study comparing ovarian reserve tested AMH, FSH, Inhibin B, and AFC in 603 women undergoing IVF and found that AMH concentrations higher than 1.4 ng/mL correlate most significantly with live births and a serum AMH above 2.0 ng/mL correlated with the best probability of live births [28]. Another study evaluated AMH in routine IVF and determined that while AMH <1.26 ng/mL is highly predictive of a decreased ovarian reserve, when utilized alone, it is not an adequate predictor [11].

Studies suggest that AMH should be confirmed with an AFC to truly be predictive. While Ficicioglu et al. stated that an AMH cutoff level of 0.25 ng/mL was the best indicator of ovarian reserve with a high sensitivity and specificity, they found this cutoff level to be a better predictor of oocyte number rather than an accurate predictor of CP [10]. This finding was also confirmed by Kwee et al. [6]. Our study confirmed this finding with a significantly increased AFC and increased AMH levels (2.9+2.4 ng/mL) that correlated to increased CP rates of 20.0% compared to 10.0% (p=0.041). 

As for ovarian stimulation, our study also indicates that a more aggressive ovarian stimulation protocol with ChmghCG with IUI may have a better probability of resulting in CP. In fact, aggressive ovarian stimulation with the ChmghCG had almost double the CP rate (32.1%) compared to the ChCG stimulation protocol.

This observational study has several limitations and strengths. One limitation is that this study was conducted at a single center. In addition, because the population studied is primarily White and is considered to be more homogenous, this could be considered both a limitation and a strength, as there are both racial and ethnic differences in AMH levels [12]. Likewise, this study population is more homogenous than some with regard to types of infertility.

## Conclusions

For IUI cycles where female partners had unexplained infertility, AMH values >2.1 ng/mL had double the CP rate compared to IUI cycles where women had AMH values <2.1 ng/mL. This rate increased even further with a more aggressive stimulation protocol (ChmghCG), resulting in a CP rate of over four times more likely when compared to IUI cycles in women with AMH values <2.1 ng/mL and with ChCG stimulation protocol. The results of this study suggest that a higher AMH and a more aggressive ovarian stimulation protocol IUI have a better probability of resulting in CPs and may therefore be useful in evaluating IUI candidates and setting forth realistic expectations. Further studies that examine larger numbers of IUI patients with AMH would better delineate the best stimulation protocol resulting in CPs and live births.
